# 1022. Detection of Mpox virus with the Cepheid Xpert Mpox assay in oropharyngeal, anorectal and cutaneous lesion swab specimens

**DOI:** 10.1093/ofid/ofad500.053

**Published:** 2023-11-27

**Authors:** Gregory Damhorst, Kaleb McLendon, Evelyn Morales, Zianya Solis, Eric Fitts, Heather Bowers, Courtney Sabino, Julie Sullivan, Morgan Greenleaf, Jonathan Colasanti, Anandi N Sheth, Boghuma K Titanji, Greg S Martin, Leda Bassit, Wilbur A Lam, Anuradha Rao

**Affiliations:** Emory University, Atlanta, GA; Emory University, Atlanta, GA; Emory University, Atlanta, GA; Emory University, Atlanta, GA; Emory University, Atlanta, GA; Emory University, Atlanta, GA; Emory University, Atlanta, GA; Emory University, Atlanta, GA; Emory University, Atlanta, GA; Emory University School of Medicine, Atlanta, Georgia; Emory University School of Medicine, Atlanta, Georgia; Emory University, Atlanta, GA; Emory University School of Medicine, Atlanta, Georgia; Emory University, Atlanta, GA; Emory University School of Medicine/Georgia Institute of Technology, Atlanta, GA; Emory University School of Medicine,, Atlanta, Georgia

## Abstract

**Background:**

The global spread of Mpox in 2022 largely affected men who have sex with men and exposures via the oropharynx and anorectum are thought to contribute to sexual transmission. New molecular platforms including point-of-care assays for detection of Mpox DNA from cutaneous lesion samples have received emergency use authorization in the United States but these technologies are not validated for use with other sample types, including oropharyngeal or anorectal swabs.

**Methods:**

Anorectal, oropharyngeal and cutaneous lesion swabs swabs were initially collected and frozen dry at -80C during clinical evaluation for suspected Mpox at a large HIV care center in Atlanta, GA. Swabs were thawed to room temperature and eluted in 3 mL of transport medium prior to testing on the Cepheid Xpert platform, which was performed according to the assay's instructions for use. DNA extracted from 140 μL of the same sample was used for non-variola *orthopoxvirus* (E9L-NVAR) PCR as a reference test and RNaseP PCR as a sample adequacy control.

**Results:**

Positive agreement between the Xpert Mpox assay and in-house PCR was 100% for 9 cutaneous lesion swabs, 8 oropharyngeal swabs, and 5 anorectal swabs (see Figure). There were no false-negative results returned by the Xpert. Overall negative agreement was 86.7% (26/30). The Xpert assay was positive in 6 specimens where viral DNA was not detected by the NVOG assay including two specimens that also did not have detectable RNAseP. 4 of these 6 specimens with discordant results were from patients with viral DNA detected at other anatomic sites.

Results of the Xpert Mpox assay, NVAR PCR, and sample adequacy controls for cutaneous lesion, oropharyngeal and anorectal specimens.

**Figure:**
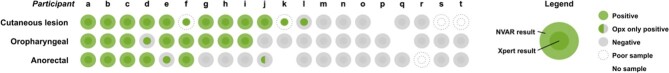

The Xpert assay was positive for 100% of specimens when the non-variola orthopoxvirus (NVAR) target detected by our reference assay and there were no false negative results. The Xpert assay also detected viral targets in 6 specimens where no viral DNA was detected by the NVAR assay. However, four of these specimens were collected from patients with Mpox DNA detected at other anatomic sites.

**Conclusion:**

The Xpert Mpox assay had perfect positive agreement with the NVAR PCR assay in anorectal, oropharyngeal and cutaneous lesion swab specimens suggesting it has high sensitivity for diagnosis of Mpox infection at the point-of-care, including with not-yet validated sample types. Specificity of the assay is difficult to estimate from our data, as a likely explanation of our results is that the Xpert Mpox assay has a lower limit of detection compared to our reference assay. This is evidenced by detection of Mpox DNA with high Ct values in samples from patients with viral DNA confirmed by both assays at other anatomic sites. The Xpert Mpox assay can be expected to perform well with oropharyngeal and anorectal swab specimens in clinical settings.

**Disclosures:**

**Jonathan Colasanti, MD, MSPH**, DKB MED LLC: Honoraria|Prime Education LLC: Advisor/Consultant

